# Updates on the role of TRIM proteins in AIDS: molecular mechanisms and potential for interventions

**DOI:** 10.3389/fimmu.2026.1742036

**Published:** 2026-04-07

**Authors:** Jingxian Chen, Siyu Chen, Yu Xu, Xinling Wang, Meixiu Jiang

**Affiliations:** 1 The Huankui Academy, Jiangxi Medical College, Nanchang University, Nanchang, Jiangxi, China; 2 School of Pharmacy, Jiangxi Medical College, Nanchang University, Nanchang, Jiangxi, China; 3 Jiangxi Province Key Laboratory of Bioengineering Drugs, The National Engineering Research Center for Bioengineering Drugs and The Technologies, Institute of Translational Medicine, Jiangxi Medical College, Nanchang University, Nanchang, Jiangxi, China

**Keywords:** advanced HIV infection, clinical perspective, HIV, mechanisms, TRIM proteins

## Abstract

AIDS (acquired immunodeficiency syndrome) is the final stage of infection with the human immunodeficiency virus (HIV) and adversely impacts the health of affected people globally, placing an added burden on healthcare systems. Nonetheless, advanced HIV infection is still not effectively curable, so the search for new drug targets remains an important research focus. Tripartite motif (TRIM) proteins constitute an extensive family of ubiquitin E3 ligases that regulate a wide range of cellular processes. Several recent studies have shown that many of TRIM proteins can take part in host defense to combat viral infection by diverse and distinct molecular mechanisms involving interaction with the NF-κB (Nuclear Factor kappa-B) pathway, JAK(Janus Kinase)-STAT (Signal Transducer and Activator of Transcription) pathway, RLR/MDA5 (Melanoma Differentiation-Associated protein 5) pathway, as well as IRF (Interferon Regulatory Factor) pathway; it can even induce premature degradation of viral proteins. Thus, this review aims to offer an in-depth insight into the roles of TRIM proteins in the pathologic progression of advanced HIV infection, especially on HIV-1 invasion and long terminal transcription inhibition and nonhistone protein reversible ubiquitination, which may afford therapeutic targets for this challenging disease.

## 1 Introduction

AIDS (acquired immunodeficiency syndrome) typically follows a pattern starting with an acute infection that may resemble mononucleosis, followed by a latent asymptomatic period (WHO Clinical Stage 1), then progressing to WHO Clinical Stages 2 to 3. WHO Clinical stage 3 conditions include unexplained manifestations, including anemia (<8 g/dl), neutropenia (<0.5 × 10^9^ per liter) or chronic thrombocytopenia (<50 × 10^9^ per liter) (1). More than 40,000,000 deaths have been estimated to date since the first case was written in 1981. According to the latest surveillance data from China CDC, as of June 30, 2025 in China there were cumulatively reported living advanced HIV infection cases for a total of 1,387471 and cumulative new deaths for a total of 506664 (2, 3). Thus, advanced HIV infection is an epidemiologic, public health, medical, social and political problem to be solved.

Antiretroviral drugs are already in use for the treatment of HIV infection. Examples of antiviral medications that may be included are nucleoside reverse transcriptase inhibitors (NRTIs), non-nucleoside reverse transcriptase inhibitors (NNRTIs), protease inhibitors, integrase inhibitors, and entry/fusion inhibitors (4). These medications slow the advancement of acquired immune deficiency syndrome (AIDS), by inhibiting reproduction of the HIV organism in our figure. But advanced HIV infection currently has no cure, to be clear. Thus, searching and identification of novel drug targets is a necessity to obtain disease treatment outcomes, and finally in order to control this deadly disease (5–13).

TRIM proteins, a heterogeneous family of proteins, play seminal roles in several cellular processes such as innate immunity and cell differentiation, development among other cellular processes. Collectively, it is emerging that TRIM proteins can modulate immune responses (innate or adaptive) through their participation in signaling pathways like NF-κB or interferon regulatory systems. In addition to their vital role in maintenance of cellular homeostasis, TRIM proteins are known to modulate host defense responses against a plethora of pathogens such as bacteria, viruses and parasites by regulating autophagy, ubiquitination and cytokine production circuits (14–18).

It has been recently reported that TRIMs have a significant role in the processes underlying antiviral responses. This review encapsulates their roles based on previous investigations, elucidating the underlying mechanisms in HIV infection and potentially providing TRIM proteins as an innovation tool for the therapeutic of advanced HIV infection.

## 2 TRIM family

### 2.1 Structure of TRIM family proteins

TRIM proteins have a highly conserved N-terminal RBCC domain made up of a RING finger domain, one or two B-box zinc finger domains and a coiled-coil domain which collectively define this family of proteins (19–23).

The typical location of the RING domain is 10–20 residues downstream from the initiating methionine. Initially, this domain was proposed to be responsible for DNA binding and recognition. The RING domain coordinates two zinc ions to form a RING finger motif, with structural similarities to classic zinc finger domains and this was established in research over the last decades. This configuration stabilizes the interaction in E2 enzymes to promote ubiquitination. It is interesting to note that the RING finger domain is a characteristic feature of a diverse range of E3 ubiquitin ligases (24).

The B-box is a zinc-binding motif found only in TRIM proteins, located C-terminal to the RING domain and able to chelate one or two zinc ions. Due to its structural similarity to the RING finger, the B-box can also mediate substrate ubiquitination. Accordingly, B-box domains are divided into type 1 (B-box1) and type 2 (B-box2). It has been proposed that the B-box1 domain functions either as an independent E3 ligase or acts to increase the catalytic activity of RING-type E3 ligases. The TRIM homology domain is also dependent on the presence of a B-box2 domain, which in some contexts appears to be helpful but may not always be required. However, it has been suggested that this structure can alter context-dependent biochemical activity by potentially influencing RING function or combining with B-box1 thus having possible upstream effects upon substrate specialization and/or E3 ligase operating efficiency (25, 26).

The coiled-coil domain usually consists of two or three helical segments whose lengths are generally about 100–200 amino acid residues1. It is well recognized that its mechanical properties are derived from a supercoiled organization of α-helices, held predominantly via hydrophobic interactions rendering its structure rope like. In TRIM proteins, this domain is responsible not only for homodimerization between similar subunits but also for heterodimeric associations with other proteins. This, in turn, determines not only their subcellular compartments towards which TRIM proteins are recruited but also the partnership patterns TRIM proteins form via these collaborations (25, 27).

**Figure 1:**
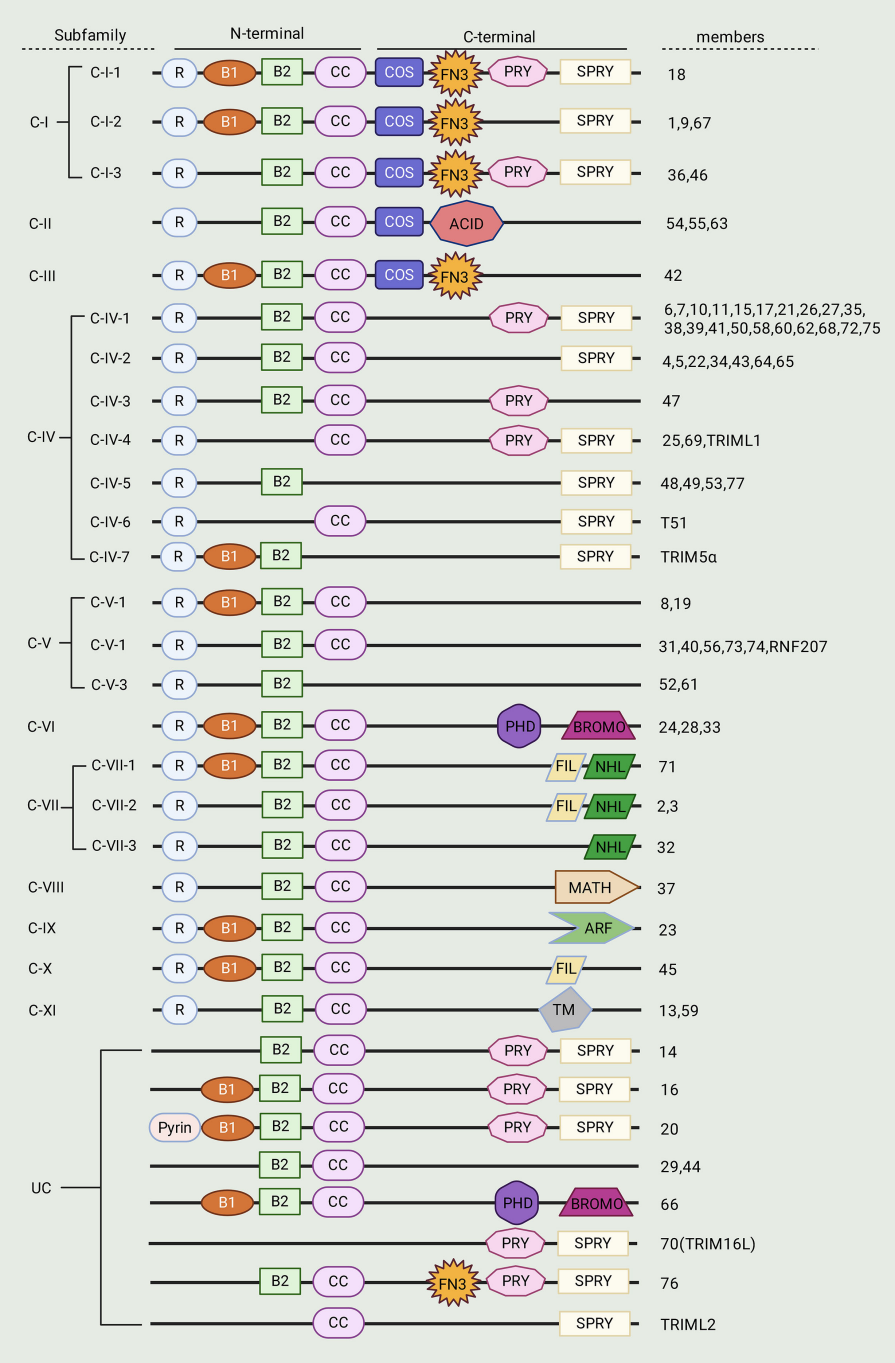
The classification of TRIM proteins based on structure. TRIM proteins are structurally categorized into 11 distinct subtypes and an unclassified subgroup (UC), which are defined by the composition of their variable C-terminal domains. A highly conserved RBCC motif, consisting of a RING finger domain, one or two B-box zinc finger domains, and a coiled-coil region, is characteristically found at the N-terminus. Key functional C-terminal domains include the PRY/SPRY (B30.2) domain, which mediates target recognition in immune regulation, as well as COS, FN3, PHD, MATH, and TM domains, each conferring specific binding and functional properties. This variability enables TRIM proteins to participate in diverse cellular processes such as ubiquitination, signaling, and transcriptional regulation. Meanwhile, the unclassified group lacks a RING-finger domain.

While all TRIM proteins have this N-terminal motif conserved, there is notable diversity in their C-terminal domains that forms the basis for their classification into 11 distinct subgroups (C-I through C-XI), as well as an unclassified subgroup (UC) (**Figure 1**) (25, 28). Among these, the PRY/SPRY (B30. 2) domain which is particularly interesting as it acts on pathogen recognition and or surface molecule binding to regulate innate immune responses and host-pathogen interactions. Further functionally relevant C-terminal domains comprise COS (C-terminal Subgroup One Signature), FN3 (Fibronectin Type III), PHD (Plant Homeodomain) and MATH (Meprin and TRAF Homology) as well as TM (Transmembrane region) domains, either endowing each member of the TRIM family with distinct binding specificities or biological roles (**Table 1**) (29). This arrangement, consisting of well-conserved N-terminal motifs grafted with variable C-terminal extensions, permits TRIM proteins to mediate versatile biological functions, such as protein ubiquitination and signaling to transcription. Interestingly, a subset of TRIM proteins that are categorized into the UC group do not possess RING domain (which is responsible for E3 ligase activity), making this family particularly functionally diverse (30).

**Table 1:** Functions of C-terminal domains of TRIM proteins.

Domain name	Full name	Function	Domain name
PRY-SPRY	PRY/SPRY domain	Protein-protein interaction, recognition of viral proteins, involvement in innate immunity	PRY-SPRY
COS	C-terminal subgroup one signature	Interaction with the microtubule cytoskeleton	COS
FN3	Fibronectin type III	Serves as a molecular scaffold, mediating protein interactions	FN3
ACID	Acid-rich region	Acidic region rich in glutamate, involved in ubiquitin-mediated degradation	ACID
FIL	Filamin-type IG domain	Regulation of the immune system and RNA binding	FIL
NHL	NHL repeats	Binding to specific RNA sequences or structures	NHL
MATH	Meprin and TRAF homology	Mediation of protein-protein interactions, formation of oligomeric structures	MATH
ARF	ADP-ribosylation factor	Possesses GTPase activity, involved in autophagy regulation	ARF
TM	Transmembrane region	Localizes to the endoplasmic reticulum, suppresses inflammatory responses	TM
PHD-BROMO	PHD-Bromodomain	Recognition of histone modifications, regulation of transcription	PHD-BROMO

### 2.2 Function of TRIM family proteins

TRIM proteins possess dual functional capabilities as they are involved in both ubiquitination and SUMOylation processes. Through their RING domains, many TRIM members function as E3 ubiquitin ligases, catalyzing the attachment of ubiquitin chains to target proteins and regulating diverse cellular pathways including innate immunity, signal transduction, and protein degradation. Concurrently, specific TRIM proteins also participate in SUMOylation—either by acting as SUMO E3 ligases themselves or by serving as SUMO-modified substrates—thereby modulating transcriptional repression, chromatin organization, and antiviral defense mechanisms. This functional duality allows TRIM proteins to integrate ubiquitin and SUMO signaling pathways, coordinating complex cellular responses through both degradative and non-degradative regulatory mechanisms.

#### 2.2.1 Ubiquitination functions of TRIM proteins

TRIMs do possess a conserved RING domain acting as an E3 ubiquitin ligase, thus forming an important core component of the ubiquitous degradation machinery known as the ubiquitin-proteasome system (UPS), which includes ubiquitin (Ub) itself, ubiquitin-activating enzymes (E1), ubiquitin-conjugating enzymes (E2), and proteases with their substrates (31, 32).

Ubiquitin, a 76‐amino acid eukaryotic protein (~8.5 kDa), acts as a degradation signal when it is assembled into chains that are recognized by the proteasome. The process of ubiquitination is initiated when E1 activates ubiquitin and forms a thioester linkage between its catalytic cysteine and the C-terminal glycine of the ubiquitin to transfer it to an E2 conjugating enzyme. E2s, which also harbor active-site cysteines, work together with E3 ligases like TRIM proteins to provide substrate specificity and execute the attachment of ubiquitin to target proteins (33–36).

Polyubiquitination occurs through chains formed with the use of one of the seven lysine (K6, K11, K27, K29, K33, K48 or k63) in ubiquitin. Different types of linkages yield diverse functional outcomes: K48-linked chains generally mark substrates for degradation by the proteasome, whereas K63-linking is usually involved in non-degradative states such as signal transduction; K6-linked chains are linked with mitochondrial homeostasis, while all other linkages (K27, K11 and K29) can also act within a proteolytic mechanism. Two ubiquitin chain types between amino acid K48 and K63 are abundant in mammals. These polyubiquitin tags are recognized by the proteasome, which decomposes the tagged protein into short peptides and recycles ubiquitin. In addition to homotypic chains, heterotypic ubiquitination enhances structural and functional diversity. K11/K48-branched ubiquitin chains, for instance, promote degradation efficiency (34, 37).

By binding substrate recognition modules located in their C-terminal regions and utilizing E3 ligase activity, TRIM proteins assemble specific ubiquitin chains and orchestrate numerous cellular processes from NF-κB and interferon signaling to p53-mediated responses, apoptosis, and antiviral defense. Certain members of the TRIM family also function as scaffolds or modifiers of protein function without provoking degradation. The odyssey of TRIM proteins illustrates their functional pleiotropy, which helps explain their involvement in numerous human diseases (38–40).

#### 2.2.2 SUMOylation functions of TRIM proteins

The RING domain of TRIM proteins is a cross-braced structural configuration formed by coordination with zinc ions. This exactly provides E2 with a structure that it can bind to. Relevant studies have shown that this complex structure promotes and catalyzes the reaction by stabilizing the E2~SUMO thioester bond to form a closed conformation. The molecular weight of the SUMO molecule is approximately 11 kDa. Its conformation has a typical βββαβαβ fold (41), and generally speaking, it is very similar to ubiquitin at the three-dimensional level. However, due to differences in surface electrostatic potential distribution and amino acid sequence composition, SUMO has unique functional properties. It obtains activity by hydrolyzing and removing the C-terminal peptide through ubiquitin-like protease 1 (ULP1) or sentrin-specific protease 1 (SENP1) (42). SUMOylation is a reversible post-translational modification. It is mediated by a hierarchical enzymatic cascade reaction, including the E1 activating enzyme (SAE1/SAE2), the E2 conjugating enzyme (Ubc9), and a series of E3 ligases (including members of the PIAS family and RanBP2). Their function is to jointly promote substrate specificity and conjugation efficiency. In essence, SUMOylation actually has two mechanisms. One is covalent modification, forming an iso peptide bond that is covalently linked to the lysine residue of the target protein. The other is to promote non-covalent interactions between SUMO-modified substrates and effector proteins that contain SUMO-interacting motifs (SIMs) (43). The SIM mentioned here generally consists of four hydrophobic residues. It can regulate substrate function and stability by binding to SUMOylated proteins (44). These will be discussed in detail later.

## 3 TRIMs in virus infection: the molecular mechanisms

TRIM proteins serve as important E3 ubiquitin ligases that globally regulate innate immunity via different mechanisms of ubiquitination. They tightly modulate important signaling pathways such as NF-κB and interferon responses (JAK-STAT, RLR/MDA5, IRF pathways, PKR pathway) by promoting proteasomal degradation through K48-linked ubiquitination or signal activation through chains K63. Moreover, TRIM proteins can directly bind and promote the degradation of viral components via ubiquitin-mediated processes, and they have established roles in triggering premature disassembly of viral capsids that also do not depend on ubiquitination. This intricately structured regulatory network empowers TRIM proteins to achieve optimum balance between antiviral defense and immune homeostasis indispensable for host protection.

### 3.1 By regulation of NF-κB pathway

The NF-κB signaling pathway is a master effector of immune and inflammatory responses, and cell survival, comprised of cytoplasmic NF-κB hetero or homodimers bound to inhibitory IκB proteins (45, 46). In response to cytokines, pathogen or stress signal, membrane receptors signal the IκB kinase (IKK) complex including IKKα, IKKβ and its regulatory subunit NEMO (NF-κB Essential Modulator) (47). IKK phosphorylates IκB, which then is K48-linked ubiquitinated and degraded by proteasome leading ultimately to the release of NF-κB for translocation into nucleus to activate target genes such as pro-inflammatory cytokines, anti-apoptotic factors and immune regulators (48, 49). In addition to its role in transcriptional regulation, NF-κB also directly inhibits viral replication through competition with viral transcription factors and induction of antiviral proteins that disrupt the assembly of viral replication complexes or mediate degradation of the entire viral RNA (50–52). However, the HIV-1 long terminal repeat (LTR) has canonical NF-κB binding motifs, and one of the effects of NF-κB activation is enhanced viral transcription, especially in activated CD4^+^ T cells and macrophages. Inducible NF-κB activity directly promotes HIV gene expression by binding to the LTR promoter, as shown in early studies (53–57). This should be attached to great importance.

**Figure 2:**
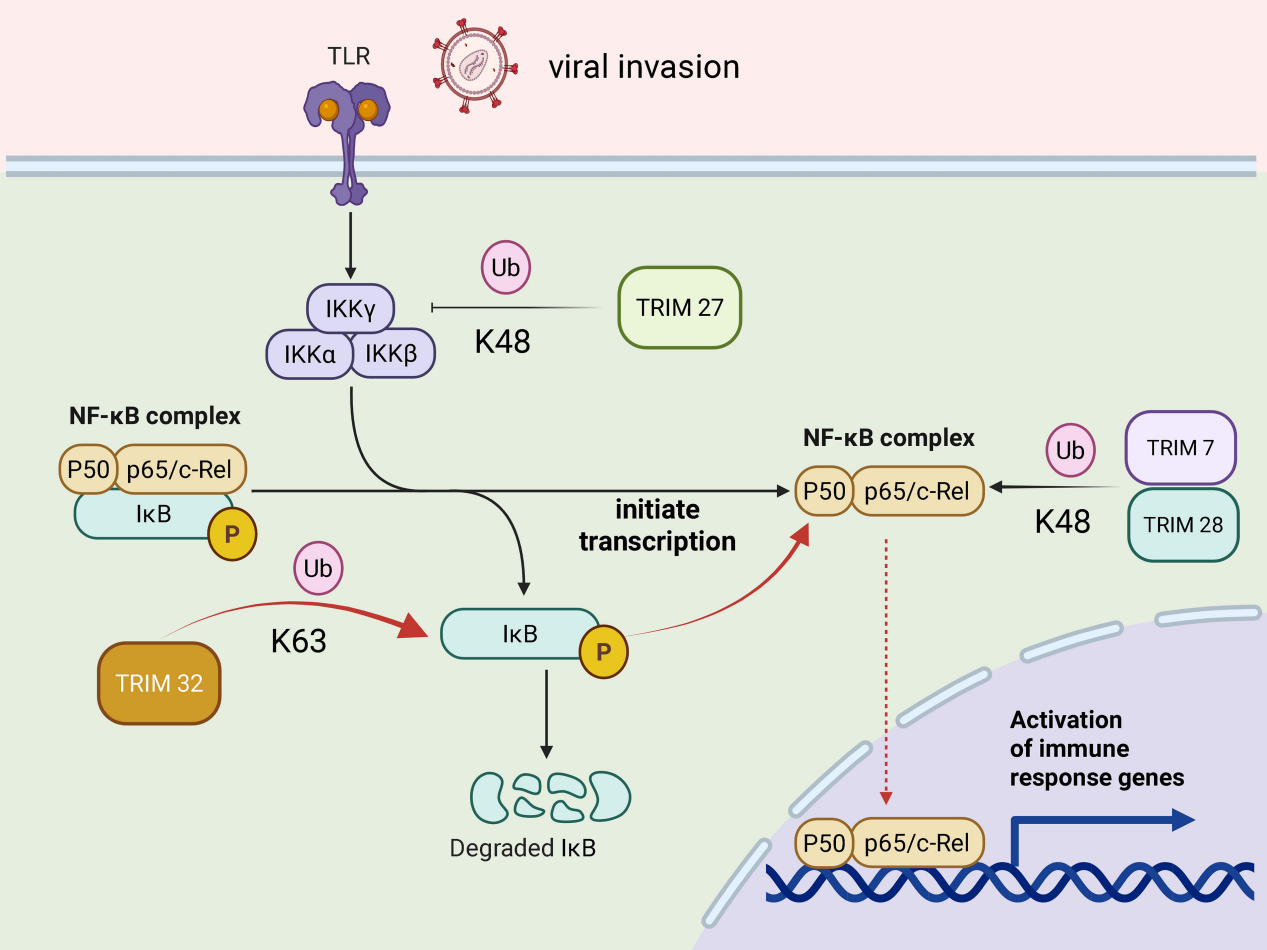
TRIM protein acts on the NF-κB pathway. Under HIV infection conditions, the mechanism of interaction between TRIM proteins and the NF-κB signaling pathway. Viral infection stimulates cell membrane receptors (TLR), resulting in the activation of the IKK complex. TRIM32 through distinct K63-linked ubiquitination mechanisms synergistically enhance NF-κB-mediated transcription, driving robust inflammatory and antiviral responses. To further phosphorylate and activate the catalytic subunit IKKβ, which then phosphorylates its canonical substrate, IκBα, triggering its K48-linked ubiquitination and proteasomal degradation. Concurrently, TRIM32 also promotes NF-κB signaling by directly mediating K63-linked ubiquitination of IκBα, which does not lead to its degradation but instead induces its dissociation from NF-κB, thereby facilitating the release and nuclear translocation of NF-κB. TRIM27 suppressed NF-κB activation, leading to a reduction in the downstream type I interferon response that promotes viral replication.

Multiple TRIM proteins regulate the NF-κB pathway via particular ubiquitination mechanisms (**Figure 2**). TRIM32 controls NF-κB activity via K63-linked ubiquitination of its substrate and modulates the generation of proinflammatory cytokines and antiviral genes. Based on its antiviral mechanism, it is speculated that its role in viral infections is context-dependent—potentiating NF-κB activation at early stage of HIV infection but eventually contributes to dampening it during later stages to minimize immunopathology (58, 59).

A second class of TRIM proteins modulates NF-κB signaling via K48-linked ubiquitination. TRIM27 suppressed NF-κB activation, leading to a reduction in the downstream type I interferon response that promotes viral replication. This function could be related to its E3 ubiquitin ligase activity by which it may enhance the ubiquitination and degradation of signal transducers in the NF-κB cascade similar to ascribed role in mediating K48-linked ubiquitination and degradation of TBK1 in IRF3 pathway (60). These interactions help maintain immune tolerance to prevent autoimmune inflammation. This can facilitate the assembly of inhibitory complexes as well as induce ASF1 (Anti-Silencing Function 1) expression, and supports antiviral defense against influenza, HSV (Herpes Simplex Virus), and HIV.

### 3.2 Regulation of interferon signaling pathways

Interferons (IFNs) are a critical aspect of the innate immune response (61). Upon viral infection, host cells produce signaling proteins that have a central role in antiviral defense called IFNs, and these alert neighboring cells to activate their antiviral mechanisms. The major pathway that mediates IFN signaling is the Janus kinase-signal transducer and activator of transcription (JAK-STAT) pathway that acts as a significant avenue for propagating an interferon signal (62). Interestingly, many TRIM proteins have been found to be key regulators of IFN-related signaling pathways. For example, while some members of the TRIM family regulate JAK-STAT signaling cascades via ubiquitination-independent mechanisms, others serve to attenuate the magnitude and persistency of interferon signatures through similar means (63). In addition to JAK-STAT, TRIM proteins are also involved in cytosolic sensing pathways, such as the RLR/MDA5 pathway: recognition of viral RNA that leads to production of IFN (64). Several TRIMs also regulate activation of IRF transcription factors in a positive or negative manner via direct interactions or ubiquitin-related regulation (65, 66).

#### 3.2.1 Regulation of JAK-STAT pathway

Cytokine signal transduction occurs predominantly via the JAK-STAT signaling pathway that is widely recognized as a central mechanism (67, 68). It starts with the binding of interferon (IFN) to its cognate receptor and the subsequent activation of JAKs that are associated with receptors. Then activated JAKs phosphorylate Signal Transducers and Activators of Transcription (STAT) proteins, the phosphorylated STAT proteins dimerize and translocate to nucleus, where they function as transcription factors to promote expression of interferon-stimulated genes (ISGs). The antiviral effectors encoded by these ISGs, for example of Mx proteins or Oligoadenylate Synthetase (OAS), act by directly inhibiting viral replication via interference with viral protein synthesis or degradation of viral RNA (69, 70).

**Figure 3:**
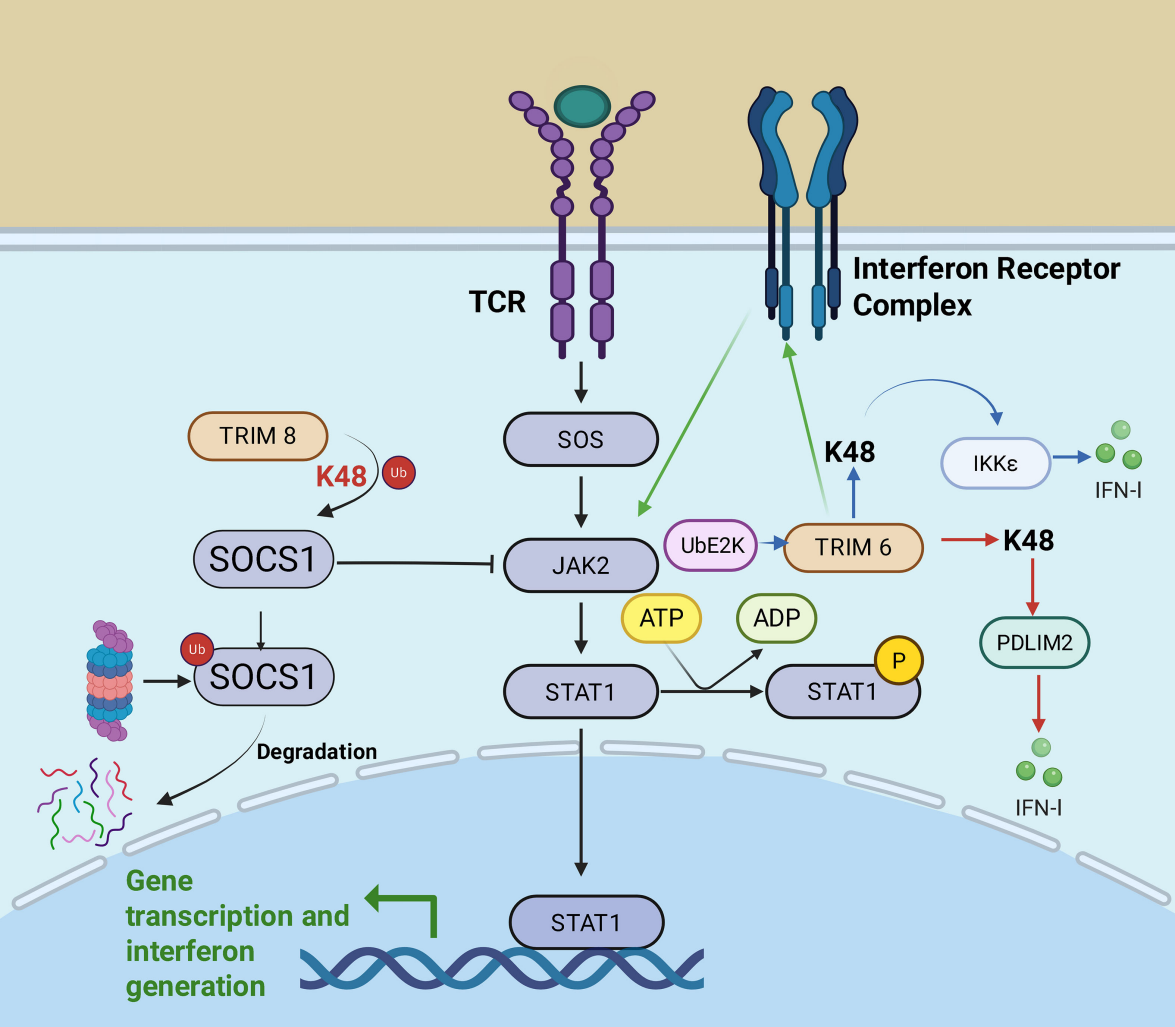
TRIM proteins exert the antiviral effects through modulation of the JAK-STAT signaling pathway. Following T cell receptor (TCR) activation, TRIM8 promotes K48-linked ubiquitination and subsequent degradation of SOCS1. As SOCS1 normally inhibits JAK2, its removal allows JAK2 to activate downstream STAT1, thereby promoting gene transcription and amplifying JAK-STAT signaling. TRIM6 enhances the JAK–STAT signaling pathway through a distinct mechanism involving the synthesis of unanchored K48-linked polyubiquitin chains in cooperation with the E2 enzyme UbE2K, which activates IKKϵ kinase to promote type-I interferon signaling and establish an antiviral state. It also targets inhibitory host factors such as PDLIM2 for K48-linked ubiquitination to fine-tune the interferon response and prevent premature signal attenuation, while directly associating with the interferon receptor complex to facilitate signal transduction and promote robust STAT1/JAK2 activation, in addition to restricting viral replication via ubiquitination of viral proteins.

A number of TRIM proteins modulate the JAK-STAT pathway through ubiquitination, often by mediating the proteasomal degradation of negatively acting JAK-STAT regulators via K48-linked ubiquitin chains (**Figure 3**). One example is TRIM8, which enhances JAK-STAT signaling in the host via binding to Suppressor of Cytokine Signaling 1 (SOCS1), an important negative regulator (70–73). TRIM8 facilitates K48-linked ubiquitination and subsequent proteasomal degradation of SOCS1, thus inhibiting SOCS1-mediated inhibition of IFN-γ signaling and facilitating the production of antiviral responses, and the resulting IFN-γ exerts direct antiviral effects that suggest a potential role for TRIM8 in suppressing HIV replication and contributing to host defense against HIV infection. In addition, TRIM8 binds directly to the IFN receptor complex and promotes its signal transduction, resulting in strong activation of STAT1 and STAT2. It also serves to ubiquitinate viral proteins which may limit replication of HIV-1 (68).

Likewise, TRIM6 also utilizes K48-linked ubiquitination to target inhibitory host factors including LIM Domain Protein 2 (PDLIM2), regulating the IFN response and repressing unwanted early attenuation of signaling. Additional mechanisms of TRIM6 in potentiating the JAK-STAT pathway are also distinct from that described here. It co-assembles unanchored K48-linked polyubiquitin chains with the partner E2 enzyme, UbE2K (Ubiquitin-Conjugating Enzyme E2 K), to then activate its own associated IKKϵ kinase, thereby enhancing IFN-I (Type I Interferon) signaling and triggering antiviral immunity, which may exert a potential anti-HIV effect through the action of interferons (74).

However, HIV-1 exerts divergent, context-dependent effects on the STAT pathway to evade immunity and maintain persistent infection. It can continuously activate STAT3 in dendritic cells and combine with STAT5 downregulation in CD8+ T cells and macrophages, which further impairs immune function. Meanwhile, STAT5 activation in CD4+ T cells enhance viral replication. Notably, HIV-1 inhibits IL-23–driven STAT3 activation in Th17 cells, which reduces IL-17 production. As a result, it disturbs the Th17/Treg balance and increases susceptibility to opportunistic infections. STAT5 additionally supports viral latency, particularly through the truncated STAT5Δ isoform. This creates more challenges for the development of therapies targeting JAK-STAT pathway (75).

#### 3.2.2 Regulation of RLR/MDA5 pathway

The pathway mediated by RIG-I-like receptors (RLRs), including key sensors such as RIG-I and MDA5, is one of the major routes for cytoplasmic detection of viral RNA (76). After binding to viral RNA, these receptors signal via the mitochondrial antiviral-signaling protein (MAVS) and exhibit a signaling cascade resulting in recruitment of transcription factors IRF3 and NF-κB leading to production of type I interferons as well as proinflammatory cytokines that will lead to inhibition of viral replication and start of immune response against virus (77).

**Figure 4:**
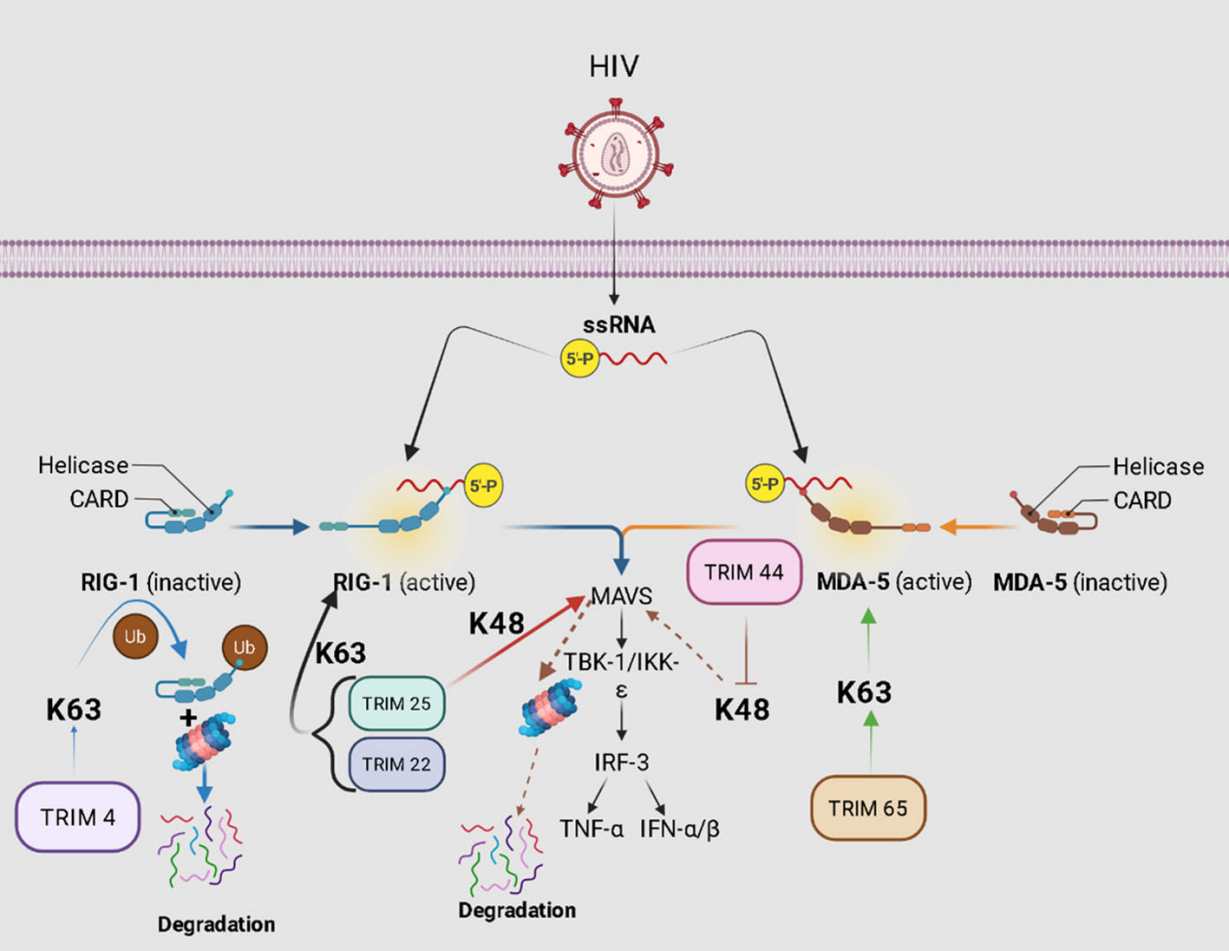
The role of TRIM proteins in the RLR/MDA5 pathway. During HIV replication, cytosolic sensors RIG-I or MDA5 recognize viral 5’-triphosphate single-stranded RNA (5’-ppp-ssRNA). This binding induces a conformational change in RIG-I/MDA5 that exposes the CARD domain, facilitating oligomerization via CARD–CARD interactions. TRIM65 specifically recognizes MDA5 and catalyzes K63-linked ubiquitination, which promotes MDA5 oligomerization and stabilizes its prion-like fibril assembly, thereby enhancing downstream signal transduction. Furthermore, TRIM22 and TRIM25 catalyze K63-linked ubiquitination of the adaptor protein MAVS, reinforcing its activation and promoting the assembly and function of the MAVS signalosome. Additionally, functioning as a deubiquitinating enzyme, TRIM44 enhances antiviral immunity by stabilizing VISA/MAVS through inhibition of K48-linked ubiquitination and proteasomal degradation—a mechanism mediated by its unique ZF-UBP domain, thereby reinforcing its regulatory role in innate immune responses. TRIM4 may enhance RIG-I activity through the conjugation of K63-linked ubiquitin chains, thereby promoting the production of type I interferons, and may also facilitate its interaction with adaptor proteins such as MAVS.

Three TRIM proteins inhibit the RLR/MDA5 pathway through ubiquitination (**Figure 4**). For example, TRIM65 amplifies MDA5-dependent antiviral signaling by mediating K63-linked ubiquitination of MDA5 to promote oligomerization and strengthen IFN production, and the resulting interferons exert broad-spectrum antiviral effects, which are speculated to include activity against HIV (78). Dynamically, TRIM22 may have possibility to amplify RLR signaling by promoting K63-linked ubiquitination of the pathways’ intermediaries like RIG-I whereas MAVS significantly potentiates interferon signal transduction thereby potentially preventing replication of HIV-1. Meanwhile, TRIM25 conjugates K63-linked polyubiquitin chains on RIG-I and promotes the K48-linked ubiquitination and proteasomal degradation of MAVS, revealing that TRIM25 plays a crucial dual role in the RLR signaling pathway, and this interaction may potentially contribute to combating HIV (38, 79–82). Additionally, TRIM44 boosts antiviral signaling by binding to and stabilizing VISA (Virus-Induced Signaling Adaptor), an essential adaptor protein in the RIG-I-like receptor pathways by antagonizing K48-linked ubiquitination leading to proteasomal decay—a feature of this module is provided through its N-terminal zinc-finger ubiquitin protease domain (ZF-UBP) that gives TRIM44 a functional advantage over classical RING domain is central for most catalytic activity. By acting as a ubiquitin stabilizer, TRIM44 reduces VISA protein degradation via this action, thus further enhancing its modulatory function in innate immune responses and potentially contributing to an anti-HIV effect. TRIM44 engages in the positive regulation of VISA to prevent its excessive turnover, resulting in a boost in the outputs from IFN-β (Interferon Beta) and TNF-(Tumor Necrosis Factor Alpha), thus enhancing antiviral innate immunity (83–85). Whereas TRIM4 acts as a counterpart that inhibits the pathway, eliciting K63-linked ubiquitination of RIG-I and targeting it to proteasomal degradation to prevent excessive immune activation, and may similarly protect against immune overactivation in the context of HIV infection (86, 87).

#### 3.2.3 Regulation of IRF pathway

A key pathway of antiviral innate immunity involves the induction of type I interferons (IFN-I) through the IRF signaling cascade. After viral infection, Pattern Recognition Receptors (PRR), like RIG-I or TLR (Toll-Like Receptor) recognize the virus components and activate downstream signaling pathways who included kinase like TBK1 (TANK-Binding Kinase 1) or IKK neighbors. These kinases phosphorylate the transcriptional factors IRF, mainly IRF3 and IRF7, which then dimerize and translocate into nuclei. In the nucleus, interferon regulatory factors (IRF) bind to interferon-stimulated response elements (ISREs) to activate IFN-I and other antiviral genes (88).

And there are of TRIM proteins that regulate the IRF pathway in a ubiquitin-dependent manner. Along similar lines, TRIM25 transcriptionally uplifts the IRF signaling cascade by stimulating K63-linked ubiquitination to activate downstream signaling pathways that include kinases like TBK1, thereby inhibiting RNA viruses such as HIV-1. (38). In addition, many other examples have been reported, which will not be listed here individually.

#### 3.2.4 Regulation of PKR pathway

Many members of the TRIM family can interact with double-stranded RNA-dependent protein kinase (PKR) to jointly resist viral infection.

HPAIV infects human lung epithelial cells. Krischuns et al. showed that during HPAIV infection, TRIM28 acts through the PKR-p38-MSK1 pathway, thereby triggering high levels of IFN-β, IL-6, and IL-8. These immune factors increase the body’s response level to pathogens, thus achieving an antiviral effect, which we speculate also includes activity against HIV (89). For TRIM25, it has two completely different roles; it can both inhibit viral infection and promote it. In most cases, it promotes type I interferon signaling by adding ubiquitin to RIG-I, which helps RIG-I bind better to MAVS, thereby promoting the body’s recognition of the virus and the downstream immune response. However, when TRIM25 is overexpressed, it prevents bcIKKe from activating antiviral genes, such as PKR, which in turn promotes replication of HIV-1 (90). TRIM21 also inhibits viruses. Relevant animal experiments show that in largemouth bass, overexpressing TRIM21 (MsTRIM21) increases the levels of IRF3, IRF7, Mx1, ISG15, PKR, and TNF-α. These immune factors can act as signals to enhance the reactivity of the immune system, strengthening the body’s immune and inflammatory responses against HIV-1 (91). In addition to functioning independently as a dsRNA kinase, PKR also directly interacts with NLRs and AIM2 to assist in the formation of inflammasomes, such as NLRP1, NLRP3, NLRC4, and AIM2 (92). These inflammasomes will all participate in downstream immune responses, raising the overall level of inflammation in the body, and may thus play a role in combating HIV.

Overall, many proteins of the TRIM family can effectively activate the immune system and increase the level of inflammatory response by regulating PKR and its related upstream and downstream pathways, thereby achieving an antiviral effect.

## 4 Treatment strategies of TRIM family proteins in HIV infection

The TRIM protein family comprises a potentially unexplored set of therapeutic HIV/HIV infection target. Of more than 70 TRIM members, TRIM5α, TRIM22, TRIM28 and TRIM37 have emerged as leading candidates on the basis of compelling preclinical evidence.

### 4.1 Targeting TRIM5α: mechanistic understanding and therapeutic approaches

Evidence is accumulating that rhTRIM5α’s restriction of HIV-1 can be modified by both capsid recognition and ubiquitin functions — importantly, its intracellular antiviral activity appears to be regulated at multiple points through the SUMO (small ubiquitin-like modifier) system (93, 94). However, the often-overlooked huTRIM5α has also shown new progress in recent years (95).

#### 4.1.1 The SUMOylation of rhTRIM5α

Many studies demonstrated that the regulation of rhTRIM5α is SUMO-dependent. First, Lys10 was identified as an SUMOylation site and showed that rhTRIM5α can be conjugated to SUMO1 and SUMO2 in cells and *in vitro*. The modification of the SUMO pathway has an impact on the efficiency of restriction: inhibition of SUMO conjugation reduces HIV-1 restriction whilst overexpression of either SUMO1 or Ubc9 increases antiviral activity. Lys10 mutation-induced loss of antiviral activity suggests that SUMOylation of rhTRIM5α is a regulatory event rather than necessary catalytic event. Interestingly, Lys10 mutation does not obliterate the antiviral activity (96–98). Under physiological conditions, rhTRIM5α is SUMOylated at Lys84 in a RanBP2-dependent manner (Recent research). As depleting the SUMO E3 ligase RanBP2 decreases rhTRIM5α SUMOylation and its ability to restrict HIV-1, it appears that SUMO modification *in vivo* is functionally relevant (99).

#### 4.1.2 SUMO-interacting motifs of rhTRIM5α

RhTRIM5α contains PIPIRs that are SUMO-interacting motifs beyond covalent modification. When partial capsid binding is retained, further disruption of these motifs dramatically decreases HIV-1 restriction, highlighting the importance for non-covalent SUMOylation dependent interactions (96). This observation is consistent with broader multiple summary studies of TRIM family proteins indicating that many function more like SUMO-dependent signaling scaffolds than conventional SUMO E3 ligases. Enhanced SIM domain binding can lead to the `integration’ of ubiquitin and SUMO signaling pathways, as well as stabilization of higher-order signaling complexes through the recruitment of SUMOylated partners (96).

Overall, the SUMO pathway, when combined with rhTRIM5α function, acts as a modulating layer that enhances rhTRIM5α activity but is not the primary effector of HIV-1 restriction; thus, this work identifies new opportunities for therapeutic manipulation of cellular antiviral defenses.

#### 4.1.3 Inducing premature viral degradation of rhTRIM5α

Induction of Premature Viral Degradation, intended early disassembling of the viral envelope limiting successful replication at the cellular level, is a host viral defense mechanism. Notably, important proteins like TRIM family members bind directly to viral capsids soon after virus entry and promote uncoating while exposing viral components to cellular degradation pathways. This leads to proteasomal or autophagic destruction, thereby stopping the viral life cycle before reverse transcription and gene expression. It is an important innate intracellular defense mechanism against diverse viruses (100, 101).

rhTRIM5α inhibits viral infection by triggering premature uncoating of the viral capsid followed by degradation (98). RhTRIM5α substantially binds to the HIV capsid through its C-terminal PRY/SPRY domain, resulting in rapid uncoating and autophagic clearance of viral particles. After binding to the capsid, rhTRIM5α also induces formation of K63-linked polyubiquitin chains and activation of the TAK1–NF-κB pathway, thus coupling direct viral restriction with innate immune signaling. It should be noted that this study was conducted based on Old World monkeys (97, 102). Deletion of this domain greatly impairs antiviral function. This mechanism is efficient against both HIV-1 and HIV-2, considering that the siRNA (Small Interfering RNA)-mediated knockdown of rhTRIM5α is responsible for restoring viral infectivity (40, 46, 103–105).

Notably, TRIM34, which has ~57% amino acid identity to rhTRIM5α, also facilitates capsid recognition and restriction in a cooperative manner. Both proteins colocalize with newly arrived viral capsids and display mutant-specific targeting. RhTRIM5α restricts the P90A HIV-1 capsid mutant, whereas TRIM34 inhibits the N74D counterpart. The TRIM34-dependent restriction of N74D is dependent on rhTRIM5α but occurs independently for P90A. These dual functional capacities provide necessary coaction for pluripotent antiviral activity against HIV and SIV in primary T cells as well as more conveniently in monocyticTHP-1 cells (106).

#### 4.1.4 Recent studies of huTRIM5α

It has been suggested that the differing patterns of retrovirus restriction by rh TRIM5α versus huTRIM5α observed in cell lines is due to variation in the SPRY v1 sequence between the two orthologues, in line with the long-standing belief that HIV-1 restriction by rhTRIM5α is species-specific (107, 108). The apparent decreased ability of huTRIM5α to restrict HIV-1 in cell lines has been associated to low affinity for the viral core and instability of the protein (109).

Compared with the proteasome-dependent mechanism observed in proteins such as rhTRIM5α, the selective autophagy mediated by huTRIM5α appears to play a more important role. HuTRIM5α can function as a platform for autophagy by forming a TRIM5α–ATG16L1–ATG5–HIV capsid complex. This complex subsequently induces the formation of an autophagosome, leading to the encapsulation of the HIV capsid. The autophagosome then fuses with the lysosome, resulting in viral degradation. In addition, huTRIM5α contains an LIR (LC3-Interacting Region) motif, which enables it to bind LC3 and p62 and thereby directly recruit the autophagic machinery (95).

Notably, the anti-HIV activity of huTRIM5α is highly dependent on cell type. Studies have shown that in epidermal Langerhans cells (LCs), HIV is captured by the Langerin receptor, which subsequently specifically recruits huTRIM5α to initiate autophagy. Langerhans cells (LCs) belong to the subset of dendritic cells (DCs) that line the mucosal epithelia of vagina and foreskin and have the ability to sense and induce immunity to invading pathogens. HuTRIM5α potently restricts HIV-1 infection of LCs but not of subepithelial DC-SIGN+ DCs. HIV-1 binding to DC-SIGN+ DCs leads to disassociation of huTRIM5α from DC-SIGN, which abrogates huTRIM5α restriction. These findings also provide new directions for potential therapeutic strategies (95, 110).

### 4.2 TRIM28: epigenetic regulation of viral latency

TRIM28 (KAP1) is a target of interest, as it promotes viral latency by epigenetically silencing the integrated provirus. Small molecule inhibitors targeting TRIM28-mediated repression are investigating as potential latency reversing agents in “shock and kill” treatment strategies. TRIM28 (KAP1) SUMOylation, required for transcriptional repression, might go beyond its ubiquitin-related activities (95, 111, 112).

#### 4.2.1 SUMO-dependent regulation of TRIM28

Recruitment of chromatin repressors that require intramolecular auto-SUMOylation of the proximal bromodomain is mediated by the PHD domain of TRIM28’s SUMO E3 ligase activity (111, 113). Although SUMOylated TRIM28 by itself directly represses transcription, it also facilitates the TRIM28–SETDB1 complex formation which is associated with heterochromatin establishment and H3K9me3 chromatin modification (114, 115). H3K9me3 enrichment promotes the binding of heterochromatin protein 1 (HP1), conferring stability to transcriptional silencing (116).

TRIM28 has specifically been identified bound at the proviral LTR in HIV-1 latency models, where it contributes to maintaining a repressive chromatin conformation characterized by high levels of H3K9me3 (117). TRIM28 depletion results in a partial reactivation of viral transcription and reduced H3K9me3 levels at the LTR, implicating its role in latency maintenance. Post-translational modifications of TRIM28, including SUMOylation have been proposed to play an important role in the stability and repressive capacity of TRIM28-containing chromatin complexes at viral promoters (111, 113, 118, 119).

Notably, TRIM28 acts as a negative regulator, prevent excessive inflammation and consequently promoting viral replication by inhibiting IRF3 activity and successive IFN-I production through K48-linked ubiquitination and degradation of IRF3 (120).

#### 4.2.2 Conclusion

These results suggest two therapeutic avenues that require further investigation. For example, inhibition of the SUMOylation pathway relieves TRIM28-mediated repression as shown here thus reactivating latent HIV whereby infected cells can then be cleared. Improvement of TRIM28 SUMOylation is another strategy that would enhance transcriptional silencing and also lead to more long-term suppression of HIV replication. From a risk-benefit perspective, the former approach may pose an unacceptably high risk considering the limited immune surveillance to which central nervous system is subjected and further limitations brought on by the blood–brain barrier. The latter strategy, however, may have greater translational value and thus warrant more research.

### 4.3 The perspective of microtubule dynamics of TRIM69

Studies have shown that TRIM69 suppresses the early stages of HIV replication in myeloid cells by regulating microtubule dynamics. Experimental evidence demonstrates that TRIM69 can directly bind to microtubules and promote the accumulation of stabilized microtubules, thereby altering cytoskeletal organization. This change interferes with the intracellular trafficking of the virus after entry into the cell. Although TRIM69 does not affect HIV-1 entry, it significantly reduces the efficiency of reverse transcription, thereby inhibiting the establishment of viral infection. Meanwhile, evolutionary analyses in primates and humans further indicate that the antiviral function of TRIM69 is highly conserved. This represents a highly meaningful new perspective, as it examines antiviral mechanisms from the standpoint of microtubule structure for the first time, thereby helping to broaden potential therapeutic strategies (121).

### 4.4 The role of other TRIMs

In contrast, PML (Promyelocytic Leukemia Protein)—a major organizer of nuclear PML bodies—displays a powerful antiviral effect through repression of viral transcription (122). Notably, TRIM19 (PML), a prototypic TRIM protein with established SUMO E3 ligase activity (123). There is close proximity between silent HIV-1 provirus and PML NBs, whereas transcriptional activation induced by TNF-α or TPA led to a significant displacement of PML NBs. PML occupancy at the HIV LTR in resting conditions and its dynamic release after induction. It contributes to viral latency (122, 124). On the other hand, it was reported that human TRIM37 which has a TRAF (TNF Receptor-Associated Factor) domain in its C-terminal domain was showed to have anti-HIV-1 activity (125). TRIM22 has diverse antiviral effects through the regulation of NF-κB and direct binding with viral RNA. It acts as a suppressor of basal HIV-1 LTR-driven transcription by preventing Sp1 binding to the HIV-1 promoter (126). TRIM11 is a new HIV-1 capsid binding protein, which restricts HIV-1 reverse transcription by accelerating viral uncoating. Overexpression of TRIM11 accelerates HIV-1 uncoating and reduces viral reverse transcription (127).

### 4.5 Potential roles of TRIMs in advanced HIV infection-associated complications

Advanced HIV infection is often accompanied by a range of complications. In this context, we also attempt to explore the potential of TRIM proteins in supporting the treatment of these complications, rather than limiting the discussion solely to the treatment of HIV infection itself.

#### 4.5.1 TRIM28 in neuroinflammation and HIV-associated neurocognitive disorder

Persistence of HIV in macrophage-lineage cells and microglia of the central nervous system causes sustained neuroinflammation associated with HIV-associated neurocognitive disorders (HAND) (128).

TRIM28 has been shown to regulate endogenous retroelement silencing and interferon-stimulated gene expression through chromatin-dependent mechanisms. TRIM28 execute structure H3K9me3-enriched heterochromatin that might modulated proviral transcription and inflammatory gene expression programs in CNS reservoirs which is regulated in SUMO-dependent manner. Recent studies in HIV neuropathogenesis speculate on the involvement of chromatin regulators, including TRIM28, as linking HIV persistence to neuroinflammatory states (118, 129, 130).

#### 4.5.2 TRIM antiviral activity and cancer

TRIM family proteins play a critical role in antiviral immunity. However, the long-term regulation of this antiviral activity may disrupt cellular homeostasis, affecting tumor suppressor pathways such as p53, and consequently altering tumor susceptibility (131). TRIM family members participate in the regulation of multiple oncogenic signaling pathways through their E3 ubiquitin ligase activity, including JAK/STAT, PI3K/AKT, TGF-β, NF-κB, Wnt/β-catenin, and p53 pathways, thereby influencing the proliferation, migration, and invasion of tumor cells (132, 133). In the early stages of tumorigenesis, TRIM proteins promote or inhibit malignant transformation by regulating ROS homeostasis (134), glucose metabolic reprogramming (135), and protein folding homeostasis (134). For example, TRIM22 induces autophagic cell death by destabilizing NRF2 and activating the AMPK/mTOR pathway (135). During tumor progression, TRIM proteins also promote tumor invasion and metastasis by regulating the epithelial-mesenchymal transition (EMT) process and maintaining cancer stem cell properties (136). Furthermore, the TRIM family plays significant roles in chemotherapy, targeted therapy, and radiotherapy resistance. For instance, TRIM7 mediates doxorubicin resistance in osteosarcoma by degrading BRMS1 (137), while TRIM23 regulates cisplatin resistance in lung cancer cells through the NF-κB/GLUT1 axis (138). In summary, when developing TRIM-based therapeutic strategies for HIV, the potential carcinogenic risks must be carefully considered.

#### 4.5.3 TRIMs in advanced HIV infection-associated opportunistic infections

As is well known, in the advanced stage, HIV causes immune system deficiency. This greatly increases susceptibility to various infectious complications and damage caused by inflammatory responses. We attempt to explore the help that TRIMs might bring in this context.

Relevant studies have found that TRIMs can play a role in tuberculosis by regulating the process of autophagy, such as TRIM16, 14, 22, 27, and 32. This inhibits bacterial proliferation. It is worth mentioning that TRIM14 and 25 prevent excessive immune responses by regulating inflammation in this process (139). This helps avoid unnecessary damage. In herpesvirus infections, experiments have proven that TRIM43 works by tagging and degrading a protein called Pericentrin. This protein can slow the replication of several herpesviruses (140). Fungal infections like candidiasis are also very common complications. Research has also pointed out the important role of TRIM26 in inhibiting excessive immune responses in these cases. It can control the production of CXCL1/CXCL2 and prevent kidney failure induced by acute nephritis (141). Human cytomegalovirus is also a common complication. Studies have pointed out that TRIM19 can limit its infection through sumoylation. In herpesvirus infections, experiments have proven that TRIM43 works by tagging and degrading a protein called Pericentrin. This protein can slow the replication of several herpesviruses (142).

However, some TRIM proteins can also promote the occurrence and development of AIDS-related complications, which requires great care when using these TRIMs to inhibit viral replication. For example, studies have pointed out that TRIM29 achieves degradation through K48 ubiquitination of the STING protein. Stimulator of interferon genes (STING) leads to the production of IFN-I and the spontaneous generation of anti-tumor CD8+ T cell responses, which are key factors in fighting HSV. This mechanism of TRIM29 undoubtedly weakens the immune response, thus giving HSV an opportunity and worsening the infection (143). Similar to this is TRIM21. In human papillomavirus (HPV) infection, TRIM21 tags and degrades the DNA sensor IFI16, blocking pyroptosis, thereby allowing the virus to evade immune system surveillance (144).

Overall, TRIM has both promoting and inhibiting effects on complications caused by AIDS. When using TRIM for antiviral therapy, we must pay attention to these aspects to prevent the effect of promoting complications from bringing negative impacts.

### 4.6 Prospects for the application of TRIM-targeted drugs in HIV

Two therapeutic strategies aim to exploit the antiviral activities of TRIM proteins: gene therapy and pharmacological modulation, with unique benefits and challenges.

#### 4.6.1 Pharmacological targeting of TRIM proteins

Unlike the aforementioned strategy, pharmacological modulation of TRIM proteins is complementary to that, and could be pursued pretty much straight away. To this end, small molecules or biologics that elevate TRIM protein expression or E3 ligase activity may potentially potentiate interferon signaling and enhance viral restriction (145) (**Table 2**). Pharmacological approaches have advantages over gene therapy with respect to their ease of administration, dose titration and reversibility. Nevertheless, they face challenges including low bioavailability, fast clearance and off-target effects caused by the structural homologues of TRIM family members. Moreover, the pharmacologically off-target binding in viral sanctuaries stills a challenge.

**Table 2:** Therapeutic targeting of TRIM proteins in HIV/advanced HIV infection.

Drug/compound	Mechanism of action	Targeted TRIM protein	Development status
cryptotanshinone	Cryptotanshinone upregulates TRIM28 expression by inhibiting HIF-1α, as HIF-1α physically interacts with TRIM28 and its activation counteracts the induction of TRIM28	TRIM28 (KAP1)	Discovery Phase
Non-immunosuppressive cyclosporine	It binds directly to cyclophilin A (CypA), preventing CypA from binding to the HIV-1 capsid and thereby allowing TRIM5α to recognize and restrict the virus	TRIM5α	Discovery Phase
Decitabine	It involves demethylation of its promoter region of TRIM37	TRIM37	Discovery Phase (In tumor models)

#### 4.6.2 Gene therapy-based strategies

Considering, the limitations of exogenous TRIM proteins expressed via plasmids, which are inefficient in providing enduring immunization to cells over extended periods of time with continuous high viral levels, gene therapy modalities have been applied that could provide an enduring endogenous expression (146). It is realized mainly by a non-viral or viral vector delivery system. Lentiviral vectors and LNPs have facilitated the development of TRIM-based gene therapy by allowing transduction of hematopoietic stem cells (HSC) and primary T-cells at high efficiencies (147). One possible solution could involve TRIM5α-engineered HSCs that generate HIV-resistant immune cell types, thereby enabling a functional cure. This strategy, however, is fraught with obstacles. A drawback of using these exogenous TRIM proteins is their potential to be recognized as foreign, leading to immune responses against the administered TRIM proteins themselves or towards vector components that can hinder long-term expression and elicit inflammatory responses. Additionally, targeting the viral reservoirs is inefficient and off-target effects or insertional mutagenesis can incur safety issues. On the other hand, the high cost and complex manufacturing processes of gene therapies will likely limit their access, especially in resource-limited regions where HIV is endemic (148).

TRIM gene function has been modulated *in vitro*, using CRISPR/Cas9-mediated genome editing. In a proof-of-concept experiment, this was demonstrated by CRISPR/Cas9-mediated disruption of TRIM5α in MDBK cells that yielded an approximately 12-fold increase in the transduction efficiency of an HIV-1-based lentiviral vector (149). Importantly, this was not an intention to provide HIV resistance as alluded from the application but rather utilizing an improvement in viral vector production titers when applied to non-human cell lines. IntroductionTRIM5α is a broad-spectrum retroviral restriction factor found in human cells, and loss of TRIM5α may undermine intrinsic antiviral defense. Knockout-based strategies are therefore not directly translatable into human therapy. Rather, these data mechanistically validate TRIM5α as a prime restriction node and provide further support for the rationale of overexpressing TRIM5α or its orthologs in human cells as an increasingly feasible anti-HIV approach (150).

While genetic editing technologies have continued to evolve, parallel efforts such as nanoparticle-based platforms for targeted delivery of antiviral effectors have also been explored. A study of SIV vaccines found that encapsulation of TLR ligands within PLGA nanoparticles provoked stronger and more lasting antibody responses in rhesus macaques, compared to alum adjuvants, and provided greater protection against vaginal challenge in animals expressing restrictive TRIM5α alleles (151). Yet, poor endosomal escape and variable encapsulation and targeting render PLGA nanoparticles substantial translational barriers to mRNA/protein delivery. Currently, lipid nanoparticles (LNPs) represent a more advanced platform than any other for TRIM-encoding mRNA delivery but findings from the adjuvant studies must be extrapolated with caution to therapeutic settings.

Recent mechanistic studies have broadened the therapeutic potential of TRIM-mediated restriction. TRIM34 has been shown to mediate a broad-spectrum lentiviral restriction in a TRIM5α-dependent manner: it shows negligible activity alone but strongly restricts the HIV-1 capsid when co-expressed with TRIM5α, suggesting functional cooperation through heterodimerization or shared downstream effectors (152). This synergy enables combinatorial targeting of functionally related TRIM paralogs. Moreover, inhibition of CSNK2—or knockdown of its downstream TRIM effectors—enhances autophagic flux and restricts diverse viruses, including HIV-1 (153). These discoveries expand the list of therapeutic targets associated with TRIM, although their relative relevance *in vivo*, tissue specificity and long-term safety will need to be systematically studied.

#### 4.6.3 Translational risks and future directions

The translational development of TRIM-targeting agents faces several common obstacles: many TRIMs, including TRIM29, exert context-dependent activities in viral restriction and oncogenesis requiring high specification to afford detrimental off-target events (154). Overzealous chronic engagement or overexpression may also invoke autoimmunity or hyperinflammation, especially in therapies having a long duration. Efficient delivery to viral reservoirs without systemic exposure is a key translational bottleneck (155), exacerbated in high burden HIV regions by economic and infrastructural shortfalls (156).

Nevertheless, future work should focus on developing tissue-specific delivery systems or highly specific small-molecule modulators with good pharmacokinetic properties, as well as biomarker development to track TRIM activity *in vivo*. Combination approaches which include TRIM-optimizing agents alongside current antiretrovirals could have synergistic potentials and limit risk of resistance emergence. Although to our knowledge no TRIM-targeting agent is yet in clinical trials for HIV, advances in protein delivery, and gene-delivery vehicles have been developed and used clinically which would be applicable within the next couple decades. Further study of TRIM biology and therapeutic targeting is a promising avenue to understand how intrinsic immunity might be recruited for long-term virologic control.

## 5 Conclusion

**Figure 5:**
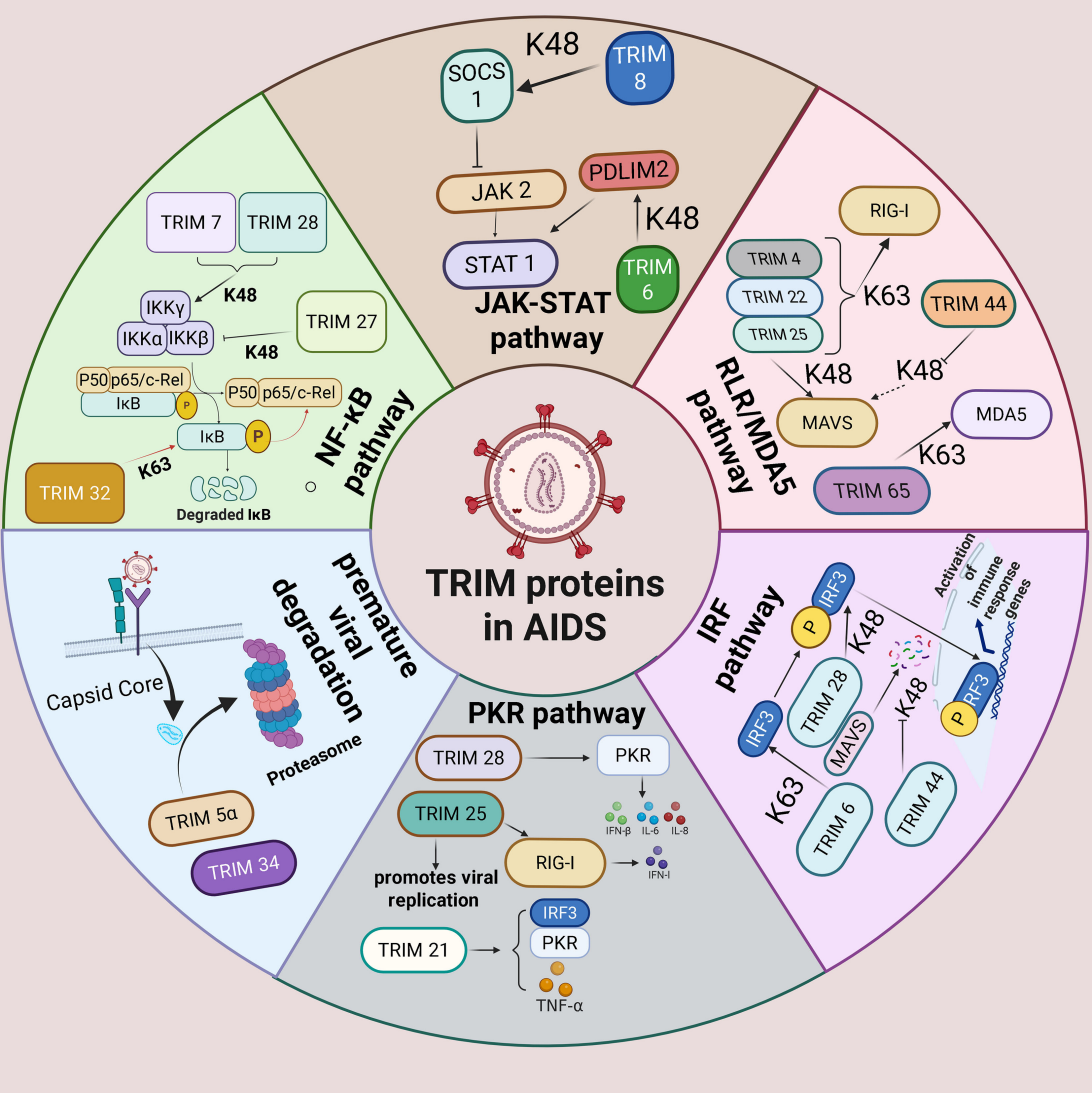
The overall graphical diagram to summarize the roles and mechanisms of TRIM proteins in AIDS. TRIM proteins mainly exert the antiviral effects via the NF-κB pathway, the JAK–STAT pathway, the RLR/MDA5 pathway, the IRF pathway, the PKR pathway, and direct viral degradation.

In summary, the present review has discussed the roles of TRIM family members in advanced HIV infection pathogenesis. Most of them mediate their antiviral functions in innate immunity and immune regulation through antagonizing specific mechanisms, including the NF-κB pathway, the JAK-STAT pathway, RLR/MDA5 pathway, IRF pathway, the SUMOylation and inducing premature viral degradation (**Figure 5**). We also briefly highlight several emerging mechanistic directions, which may provide readers with a different perspective. Additionally, we discussed the pharmacological properties of TRIM target proteins and how targeting these proteins can represent novel therapeutic strategies which could encompass antiviral and immune modulation to addressing one of patients’ major challenges: viral reservoirs.

However, there are significant barriers to the translational use of these discoveries. As such, the functional pleiotropy of TRIM proteins, as well as the possibility for off-target effects and context-dependent functions of certain family members highlight a need for highly specific intervention strategies. Future exploration in this area starts with systematic CRISPR-Cas9 screens to determine what TRIM proteins are non-redundant for restriction of HIV in primary human cells, and whether we can develop specific small-molecule agonists or antagonists targeting signaling pathways upstream or downstream of key TRIM players (TRIM5α and TRIM22 exemplified here), determining their effectiveness/side effects in advanced models including humanized mice infected with HIV.

Nonetheless, as summarized here, the evidence concludes that harnessing TRIM proteins’ antiviral arsenal presents remarkable potential for a new class of therapeutics. If this line of research continues to be pursued and focused on, it could help achieve a functional cure for advanced HIV infection.

## Glossary

ADVANCED HIV INFECTION: Acquired Immunodeficiency Syndrome
ARC: AIDS-Related Complex
ARF: ADP-Ribosylation Factor
ASF1: Anti-Silencing Function 1
CARD: Caspase Activation and Recruitment Domain
COS: C-terminal Subgroup One Signature
CRISPR-Cas9: Clustered Regularly Interspaced Short Palindromic Repeats - CRISPRAssociated Protein 9
ECSIT: Evolutionarily Conserved Signaling Intermediate in Toll pathways
FN3: Fibronectin Type III
HAND: HIV-Associated Neurocognitive Disorders
HIF-1α: Hypoxia-Inducible Factor 1 Alpha
HIV: Human Immunodeficiency Virus
HSCs: Hematopoietic Stem Cells
HSV: Herpes Simplex Virus
IFN(s): Interferon(s)
IFN-I/IFN-β: Type I Interferon/Interferon Beta
IKK: IκB Kinase
IRF: Interferon Regulatory Factor
ISGs: Interferon-Stimulated Genes
ISREs: Interferon-Stimulated Response Elements
JAK: Janus Kinase
KAP1: KRAB-associated Protein 1 (synonym for TRIM28)
LNPs: Lipid Nanoparticles
MAVS: Mitochondrial Antiviral-Signaling Protein (also known as VISA)
MATH: Meprin and TRAF Homology
MDA5: Melanoma Differentiation-Associated protein 5
NEMO: NF-κB Essential Modulator (IKKγ
NHL: NHL repeats (named after Ncl-1, HT2A, and Lin-41)
NNRTIs: Non-Nucleoside Reverse Transcriptase Inhibitors
NRTIs: Nucleoside Reverse Transcriptase Inhibitors
OAS: Oligoadenylate Synthetase
PDLIM2: PDZ and LIM Domain Protein 2
PHD: Plant Homeodomain
PML: Promyelocytic Leukemia Protein
PRY/SPRY: Domain found in TRIM proteins (also known as B30.2)
RBCC: RING-B-box-Coiled-Coil
RIG-I: Retinoic acid-Inducible Gene I
RLR: RIG-I-like Receptor
siRNA: Small Interfering RNA
SIV: Simian Immunodeficiency Virus
SOCS1: Suppressor of Cytokine Signaling 1
STAT: Signal Transducer and Activator of Transcription
TBK1: TANK-Binding Kinase 1
TCR: T Cell Receptor
TLR: Toll-Like Receptor
TNF-α: Tumor Necrosis Factor Alpha
TM: Transmembrane Region
TRAF: TNF Receptor-Associated Factor
TRIM: Tripartite Motif
Ub: Ubiquitin
UbE2K: Ubiquitin-Conjugating Enzyme E2 K
UPS: Ubiquitin-Proteasome System
VISA: Virus-Induced Signaling Adaptor (synonym for MAVS)
ZF-UBP: Zinc-Finger Ubiquitin Protease domain.
